# The positive effect of pulse electromagnetic field therapy on pain and disability in chronic low back pain: a comparative study

**DOI:** 10.1007/s00296-024-05645-x

**Published:** 2024-06-25

**Authors:** Gülşah Yaşa Öztürk, Ayşegül Yetişir, Burhan Fatih Kocyigit

**Affiliations:** 1 Faculty of Medicine, Department of Physical Medicine and Rehabilitation, University of Health Sciences, Adana City Training and Research Hospital, Adana, Türkiye; 2 Faculty of Medicine, Department of Physical Medicine and Rehabilitation, Division of Rheumatology, Çukurova University, Adana, Türkiye

**Keywords:** Low back pain, Magnetic field therapy, Physical therapy modalities, Disability

## Abstract

**Background:**

Low back pain that lasts longer than three months is called chronic low back pain. Chronic low back pain is among the most common problems in the world, causing severe disability and loss of employment in patients.

**Objective:**

To investigate the effect of pulse electromagnetic field therapy (PEMFT) added to routine physical therapy on pain and functional status in patients with chronic low back pain.

**Methods:**

This retrospective comparative study included 69 patients with chronic low back pain. The patients were divided into two groups: those who received lumbar transcutaneous electrical nerve stimulation, infrared, and ultrasound treatments, and those who additionally received PEMFT. The files of patients with chronic low back pain were reviewed, and those who had been evaluated using the Quebec Back Pain Disability Scale (QBPDS) in terms of functional capacity and effects of low back pain and the Visual Analogue Scale (VAS) for pain both before and after treatment were included in the study.

**Results:**

No significant difference was detected between the two groups’ pretreatment VAS and QBPDS scores (*p* > 0.05). The second-and-third measurement scores of both groups were significantly lower than their first-measurement VAS and QBPDS scores (p ˂ 0.001), but there was no significant difference between their second- and third-measurement scores (*p* > 0.05). According to the inter-group comparison of the VAS and QBPDS scores, the second and third-measurement scores of the PEMFT group were significantly lower than those of the control group (p ˂ 0.001).

**Conclusions:**

PEMFT seems to be able to alleviate pain intensity and ameliorate disability in patients with chronic low back pain. PEMFT can be considered an effective and safe option that can be added to routine physical therapy modalities for relieving chronic low back pain frequently encountered in clinical practice. Further studies validating the effectiveness of PEMFT could strengthen its position in the management of chronic low back pain.

**Supplementary Information:**

The online version contains supplementary material available at 10.1007/s00296-024-05645-x.

## Introduction

Low back pain is defined as pain, muscle tension, and stiffness in the area between the lower border of the 12th rib and the lower gluteal fold on the proximal thigh, with or without accompanying leg pain. Chronic low back pain, described as low back pain that lasts more than three months, is a widespread health issue around the world, imposing a significant medical and financial cost on society as a whole [1, 2]. The prevalence of chronic low back pain is 23%, and the rate of disability due to low back pain is reported to be 11–12% [3]. Low back pain imposes a tremendous burden on countries’ economies and healthcare systems due to costly treatment options and workforce loss [4]. It negatively affects the quality of life and causes significant physical and psychological health problems [5, 6]. Most cases of low back pain are of mechanical origin [7]. A multidisciplinary approach is generally required to treat chronic low back pain. Various physical therapy methods improve the functional state [8].

Electromagnetic field therapy (EMFT) has experienced a substantial surge in popularity within rehabilitative settings. This therapy offers a harmless, secure, and straightforward approach to directly address the location of injury [9]. Similar analgesic and antinociceptive effects to opiates have been attributed to pulsed EMFT (PEMFT); however, the physiological and metabolic processes by which PEMFT alleviates pain remain unclear [10]. Potential mechanisms include enhancing tissue oxygenation, vasodilation, and accelerating lymphatic circulation. These processes facilitate the decrease of pain and have anti-inflammatory and anti-edema effects. Additionally, it aids in the regeneration and repair process by boosting the synthesis of growth factors [11, 12].

While different studies in the literature endorse the use of PEMFT for low back pain management, notable issues need to be addressed. These factors encompass heterogeneity between articles, protocol discrepancies, potential bias, and variety in methodological quality [13, 14]. Therefore, there is still a need for further studies to support the efficacy of this treatment method based on a high level of evidence. Our study aimed to investigate the effect of adding PEMFT to conventional physical therapy modalities on improvement in pain and disability scales in patients with chronic low back pain.

## Material and method

### Study design

This is a retrospective comparative study. Data were collected by retrospective review of the files of patients admitted to physical therapy and rehabilitation outpatient clinics between January 2022 and June 2022.

### Study population and sample

The study comprised individuals who were at least 18 years old and had been diagnosed with chronic low back pain. These individuals were engaged in a program for physical therapy and rehabilitation. The exclusion criteria were as follows: having attended any physical therapy program within the last six months and the presence of peripheral vascular disease, rheumatic diseases, diabetes mellitus, osteoporotic fracture, acute trauma to the lumbar region, a history of fracture or spine surgery, lower extremity neurological deficits, polyneuropathy, spondylodiscitis, soft tissue or bone infections, spinal abscess, pregnancy, breastfeeding, epilepsy, a pacemaker, or hematological disorders.

Patients enrolled in a physical therapy and rehabilitation program were divided into two groups. The first group (control group) underwent lumbar transcutaneous electrical nerve stimulation (TENS) lasting 20 min, with 20 min of infrared and 5 min of ultrasound treatment. The second group (intervention group) received an additional treatment regimen involving PEMFT for 20 min (in addition to the standard regimen). The treatment protocols were scheduled for five consecutive days each week and spanned three weeks (15 sessions). The same physiotherapist provided physical therapy and rehabilitation procedures. The same physiatrist diagnosed all patients with chronic low back pain.

For the sample calculation, the study conducted by Hattapoğlu et al. [15] was taken as a reference. The number of participants required for the repeated measurements of the intervention and control groups was calculated with the WSSPAS software using the data from the reference publication [16]. Accordingly, at a power of 0.90 and a significance level of 0.05, the required sample size was 28. A total of 69 patients (35 in the intervention group and 34 in the control group) who met the criteria were included in the study.

### Data collection tools

The demographic data, including age, sex, weight, height, and comorbities were screened. The patients’ Visual Analogue Scale (VAS) scores were used to assess chronic back pain levels, and their Quebec Back Pain Disability Scale (QBPDS) scores were used to evaluate disability.

#### Visual analogue scale

The VAS score is determined by the point on a 10-centimeter line that each patient is asked to mark to indicate the level of pain they are experiencing. One end of the line represents “no pain,” and the other end represents “most intolerable pain.” The VAS score is recorded as the point that is marked [17].

#### Quebec back pain disability scale

In this index, the functional capacity of patients with low back pain and their rates of disability in activities of daily living are evaluated with a test consisting of 20 items. The difficulty of each activity is rated from 0 (“not difficult at all”) to 5 (“unable to do”). A total score of 0 indicates no problem, while 100 indicates the highest disability. The validity and reliability analyses of the scale were performed by Melikoğlu et al. [18].

The assessments were conducted by the same physiatrist before therapy, the third week after treatment, and the twelfth week after treatment.

### Statistical analysis

The data was analyzed using IBM SPSS Statistics version 25.0 (IBM, Armonk, NY, USA). The descriptive data analysis expressed frequency distributions as numbers and percentages. The Kolmogorov-Smirnov test was conducted to check the normality of the data distribution and mean and standard deviation values were used for data that conformed to the normal distribution, as well as median and interquartile range values for those without a normal distribution. The independent-sample t-test was used to analyze data with a normal distribution, and the Mann-Whitney U and Friedman test was used to analyze non-normally distributed data. The Wilcoxon signed-rank test was conducted to determine which groups caused significant differences in repeated measurements. The Chi-Square test was used in the analysis of categorical data. The effect size formula used in the Mann-Whitney U test is below [19].

*r =* ZNobs *(0.1 ≤ r ˂0.3, small effect; 0.3 ≤ r ˂0.5, moderate effect; and 0.5 ≤, large effect)*.

In statistical tests, *p* < 0.05 was considered significant.

### Ethical considerations

The study was approved by the hospital’s Clinical Research Ethics Committee, with a decision number 1829, and conducted in accordance with the principles of the Declaration of Helsinki.

## Results

The sample consisted of 69 patients, 34 in the control group and 35 in the PEMFT group. The mean age of the patients was 49.2 ± 12.7 years. The age ranges of the patients were 20–76 years in the control group and 25–67 years in the PEMFT group. Twenty-three of the patients with comorbidities had hypertension. There was no significant difference between the groups regarding age, sex, body mass index, or comorbidities. Table 1 shows the baseline characteristics of the groups.

**Table 1:** Baseline characteristics of the groups

		Control Group	PEMFT Group	*P* value
**Age**, median (IQR)		51.5 (17)	49 (16)	0.435^a^
**BMI**		27.5 ± 3.1	26.6 ± 3.9	0.262^b^
**Sex**, n (%)	Male	13 (38.2)	19 (54.3)	0.273^c^
	Female	21 (61.8)	16 (45.7)	
**Comorbidity**, n (%)	no	20 (58.8)	26 (74.3)	0.268^c^
	yes	14 (41.2)	9 (25.7)	
**Total**		34 (100.0)	35 (100.0)	

^a^Mann-Whitney U test; ^b^Independent-samples t-test; ^c^Chi-Square test; BMI: body mass index; PEMFT: pulsed electromagnetic field therapy; n : number; %: percentage; IQR: Interquartile range

There was no significant difference between the pretreatment VAS scores of the two groups. Both groups’ second- and third-measurement scores were significantly lower than their first-measurement scores (p ˂ 0.001). There was no significant difference between the second and third measurements (*p* > 0.05). The inter-group comparison of the VAS scale scores revealed that the -second and -third measurement scores were significantly lower in the PEMFT group than the control group (p ˂ 0.001). The effect size of the difference was found to be large.

**Fig. 1:**
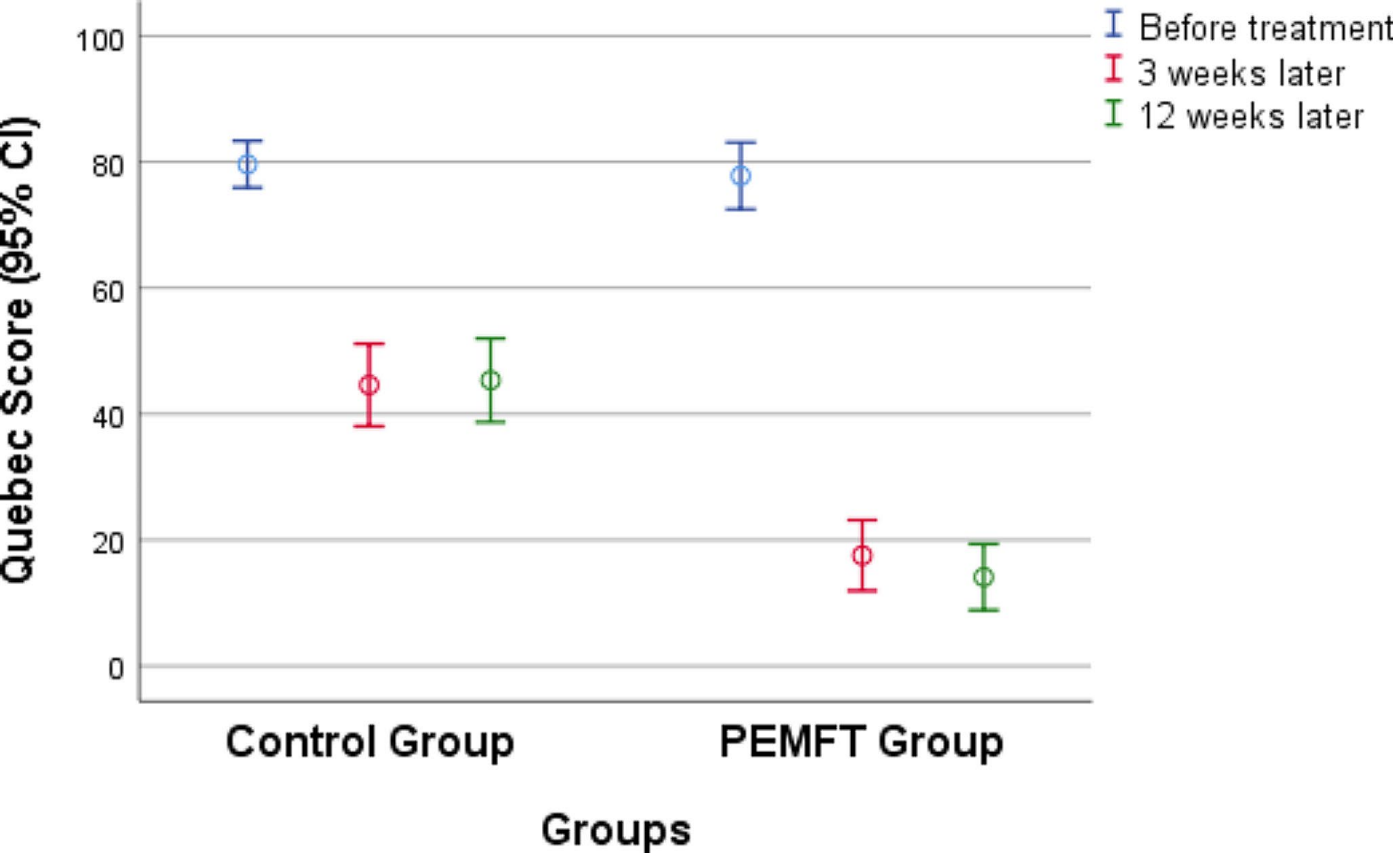
Changes in Quebec Back Pain Disability Scale scores according to groups VAS: Visual Analogue Scale; PEMFT: pulsed electromagnetic field therapy

**Fig. 2:**
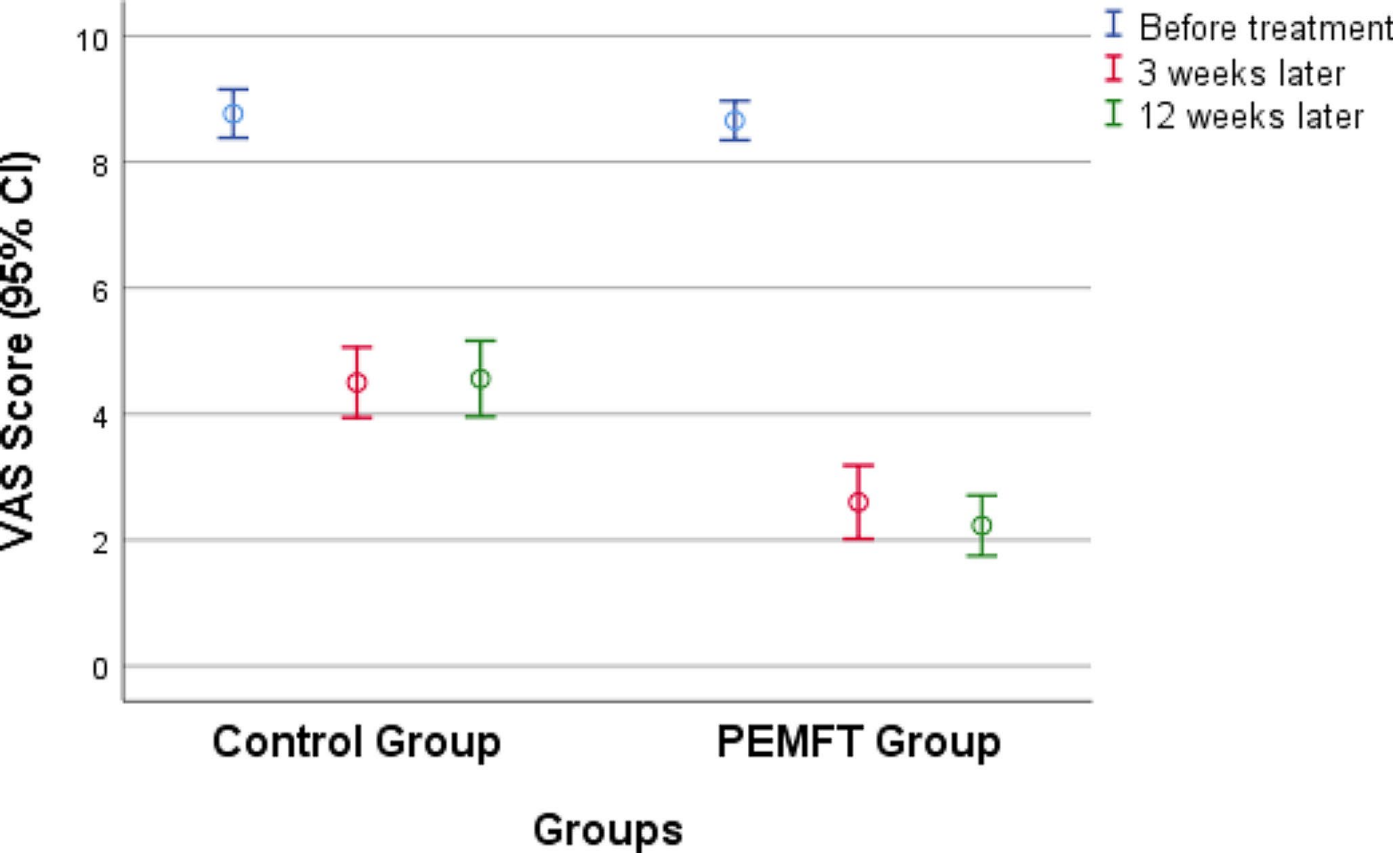
Changes in Visual Analogue Scale scores according to groups VAS: Visual Analogue Scale; PEMFT: pulsed electromagnetic field therapy

No significant difference was detected between the pretreatment QBPDS scores of the two groups (*p* > 0.05). Both groups’ second- and third-measurement scores were significantly lower than their first-measurement scores (p ˂ 0.001), but there was no significant difference between their second- and third-measurement scores (*p* > 0.05). According to the inter-group comparison of the QBPDS scores, the second- and third-measurement scores of the PEMFT group were significantly lower than those of the control group (p ˂ 0.001), and the effect size of the difference was large. Table 2 shows the VAS and QBPDS scores of the groups, and Figs. 1 and 2 present the variation in these scales according to the groups.

**Table 2:** Comparison of the VAS and QBPDS scores of the groups

		Control group	PEMFT group	*P* value	Effect size (*r*)
		Median (IQR)	Median (IQR)		
**VAS score**	Before treatment	9 (2)	9 (1)	0.529	0.075
	3 weeks after treatment	5 (2)	2 (1)	**˂0.001**	0.552
	12 weeks after treatment	5 (2)	2 (1)	**˂0.001**	0.633
	*P* value^a^	**˂0.001**	**˂0.001**		
**QBPDS score**	Before treatment	80 (11)	80 (10)	0.803	0.030
	3 weeks after treatment	50 (36)	10 (11)	**˂0.001**	0.626
	12 weeks after treatment	50 (30)	10 (8)	**˂0.001**	0.687
	*P* value^a^	**˂0.001**	**˂0.001**		

^a^Friedman test; VAS: Visual Analogue Scale; QBPDS: Quebec Back Pain Disability Scale; PEMFT: pulsed electromagnetic field therapy, IQR: Interquartile range

## Discussion

In the USA, the annual prevalence of low back pain in the adult population is 10–30%, and the lifetime prevalence of this condition is 65–80%. Treating chronic low back pain may include multidisciplinary and multimodal medical, psychological, physical, and interventional approaches. Treatment options include pharmacological, psychological, physical, and rehabilitation treatments, complementary and alternative medicine approaches, and minimally invasive percutaneous approaches [20]. In the current study, we aimed to compare the efficacy of PEMFT added to conventional physical therapy by forming intervention and control groups.

In this study, the control and PEMFT groups were similar in age, sex, body mass index, and comorbidities. The pretreatment VAS and QBPDS scores of the two groups were also similar. There are many studies in which physical therapy agents have been used in low back pain. Kim et al. [21] applied six sessions of ultrasound, TENS, interference flow, and hot pack therapy to patients with low back pain. They observed a significant decrease in the VAS, Functional Rating Index, and McGill Pain Questionnaire scores at the end of treatment compared to the pretreatment evaluation. In another study, patients with chronic low back pain underwent conventional physical therapy consisting of 20-minute TENS and hot pack therapy, followed by five-minute therapeutic ultrasound therapy, and their pretreatment Oswestry Disability Index (ODI) and VAS scores ​​were reported to significantly decrease one week and 12 weeks after treatment [22]. Similarly, in this study, the post-treatment VAS and QBPDS scores of the control group receiving conventional physical therapy were found to be statistically significantly lower than the pretreatment scores, but there was no significant difference between the third-week and twelfth-week scores. These results suggest that a conventional physical therapy regimen consisting of TENS, infrared, and ultrasound can be an effective option in the management of chronic low back pain.

In a prospective randomized controlled study comparing the effects of conventional non-invasive treatment modalities, Elshiwi et al. [23] included 50 patients with chronic nonspecific low back pain to investigate the efficacy of 50 Hz, 20 Gauss low-intensity PEMFT. The authors applied 15 min of TENS, five minutes of pulse ultrasound, and an exercise program in each session as conventional physical therapy. The first group (*n* = 25) received conventional physical therapy and PEMFT (20 min per session), and the second group (*n* = 25) received conventional physical therapy and a placebo PEMFT for a total of 12 sessions. The addition of PEMFT to conventional physical therapy resulted in superior clinical improvement in pain, functional disability, and lumbar joint range of motion in patients with nonspecific low back pain. Another randomized, double-blinded, placebo-controlled pilot study presented the results of 25 patients who were divided into the groups of usual care, PEMFT and usual care, and sham PEMFT. Significant improvements were reported in the ODI scores of the PEMFT group compared to the sham group from the baseline to the sixth week, which continued through the 12th -week follow-up [24]. In a study by Teresa et al. [25], low-energy PEMFT was found to improve neuropathic pain and functional status in chronic low back pain with a neuropathic component, compared to the control group. In a systematic review and meta-analysis of 14 studies, PEMFT significantly reduced low back pain compared to a placebo and other treatments. However, despite a significant reduction in chronic low back pain, this treatment was found to be ineffective in relieving acute pain [26]. In another study evaluating 51 patients with chronic low back pain in three groups, namely pulsed high-intensity laser (HILT), PEMFT, and control, Abdelbasset et al. [27] found significant differences in the PEMFT group in terms of the VAS, modified ODI, and pain disability index scores, as well as the range of motion at flexion. However, according to the inter-group comparison, the results of the HILT group were significantly better than those of the PEMFT group. Another study evaluated the PEMFT + therapeutic exercise and sham PEMFT + therapeutic exercise groups in chronic low back pain patients. The intervention group showed faster improvement in both pain and disability than the control group. There was a significant reduction in pain and disability scores at the end of the third week in the intervention group and at the end of the sixth week in the control group. In both groups, the improvements continued to be observed at the sixth, ninth, and thirteenth weeks, and the results of the two groups were similar at these weeks. The authors concluded that PEMFT reduced pain and disability in chronic low back pain, but it was not superior to other treatments [28].

In the current study, the post-treatment VAS and QBPDS scores of the PEMFT group were statistically significantly lower than the pretreatment measurements. However, there was no significant change in either score from the 3rd to the 12th week. When compared between the groups in the 3rd and the 12th week, the VAS and QBPDS scores were statistically significantly lower in the PEMFT group. Both groups benefited from the treatments, but the PEMFT group showed more significant improvements. The current results suggest that adding PEMFT treatment to the conventional physical therapy regimen yields favorable outcomes regarding pain and disability scores. Therefore, PEMFT can be regarded as an effective option for managing chronic low back pain in appropriate individuals.

This article has some limitations. First, the sample size is limited. Follow-up time is restricted, and long-term outcomes cannot be addressed. There is a risk of bias due to retrospective design. The data are based on self-reported scales; no performance measurements or range of motion assessments were undertaken. The article lacks scales measuring the quality of life, depression, or anxiety. The study was not planned with a randomized and prospective design. Subgroups of chronic low back pain were not evaluated. All patients were analyzed in the same pool. The neuropathic pain component was not assessed.

## Conclusion

PEMFT has shown the capacity to reduce pain intensity and enhance disability in chronic low back pain. The study found that both groups had reduced pain and disability scores following treatment, indicating the efficacy of traditional physical therapy. However, PEMFT seems to provide beneficial effects when added to a standard treatment. While the results are promising, it is important to note that the findings are not conclusive. Future studies with larger sample sizes, randomized controlled designs, longer follow-up periods, and comprehensive outcome measurements are needed to better understand the efficacy and mechanisms of PEMFT in the management of chronic low back pain. Overall, the findings of this study support the use of PEMFT as an important option in the comprehensive management of chronic low back pain, providing clinicians with another tool to improve patient outcomes and quality of life.

### Electronic supplementary material

Below is the link to the electronic supplementary material.

Supplementary Material 1

Supplementary Material 2

Supplementary Material 3

Supplementary Material 4

Supplementary Material 5

Supplementary Material 6

## Data Availability

The datasets gathered during the preparation of this manuscript are available from the corresponding author upon reasonable request.
